# Magnetic induction dependence of Hall resistance in Fractional Quantum Hall Effect

**DOI:** 10.1038/s41598-018-31205-y

**Published:** 2018-08-24

**Authors:** Tadashi Toyoda

**Affiliations:** Department of Physics, Tokai University, 4-1-1 Kitakaname, Hiratsuka-shi, Kanagawa 259-1292 Japan

## Abstract

Quantum Hall effects (QHE) are observed in two-dimensional electron systems realised in semiconductors and graphene. In QHE the Hall resistance exhibits plateaus as a function of magnetic induction. In the fractional quantum Hall effects (FQHE) the values of the Hall resistance on plateaus are *h*/*e*^2^ divided by rational fractions, where −*e* is the electron charge and *h* is the Planck constant. The magnetic induction dependence of the Hall resistance is the strongest experimental evidence for FQHE. Nevertheless a quantitative theory of the magnetic induction and temperature dependence of the Hall resistance is still missing. Here we constructed a model for the Hall resistance as a function of magnetic induction, chemical potential and temperature. We assume phenomenological perturbation terms in the single-electron energy spectrum. The perturbation terms successively split a Landau level into sublevels, whose reduced degeneracies cause the fractional quantization of Hall resistance. The model yields all 75 odd-denominator fractional plateaus that have been experimentally found. The calculated magnetic induction dependence of the Hall resistance is consistent with experiments. This theory shows that the Fermi liquid theory is valid for FQHE.

## Introduction

The basic mechanism of the integer QHE (IQHE)^1,2^ and FQHE^3,4^ is non-uniform distribution of electron density caused by the Lorentz force acting on the electrons^5^. Theoretically the non-uniform distribution can be taken into account by using the method of subsystem^6–10^, in which the system is divided into many strips of rectangular-shaped subsystems parallel to the direction of the bias current. The electron density in each subsystem may be different, but the chemical potential takes the same value. In each subsystem we derive the relation between the bias current and the transverse potential difference using the many-electron quantum field theory^11–13^. The dynamics of the electrons is described in terms of the second quantised field operators that satisfy the equal-time anti-commutation relations. The Lorentz force acting on the electrons can be calculated by the Heisenberg equations for the mechanical momentum of the electrons. We assume a model Hamiltonian for the electrons in each subsystem to be $H={H}_{0}+{H}_{{\rm{spin}}}+{H}_{{\rm{e}}}+{H}_{{\rm{int}}}$, where *H*_0_ is the kinetic energy term with the external perpendicular magnetic field, *H*_spin_ is the Zeeman spin term, *H*_e_ is the coupling to the electric field, and *H*_int_ is the electron-electron interaction term. Calculating the statistical ensemble average of the Heisenberg equations for the mechanical momentum and assuming the steady state condition, we obtain the bias current as a function of Hall voltage in each subsystem. Assuming that the statistical ensemble average of the electron number density is given by the Fermi distribution function^11^ and calculating the sum of the bias currents of all subsystems, we obtain the inverse of Hall resistance

1 $${R}_{{\rm{H}}}^{-1}=ec{B}^{-1}\sum _{q}D(q){\{1+\exp [({\varepsilon }_{q}-\mu )/{k}_{B}T]\}}^{-1},$$

where *c*, −*e*, *B*, *μ*, *k*_*B*_, and *T* are the speed of light, electron charge, magnetic induction, chemical potential, Boltzmann constant, and temperature, respectively. The single-electron energy spectrum is denoted by *ε*_*q*_ with a quantum number *q*. The degeneracy of an energy level *q* is denoted by *D*(*q*). It should be noted that the effects of the electron-electron interaction on *ε*_*q*_ can be rigorously calculated using the finite-temperature generalised Ward-Takahashi relations^12^.

## Model of FQHE

To construct a model of the FQHE let us first examine the theoretical mechanism of plateaus in IQHE, which can be quantitatively explained by adopting the Landau level ${\varepsilon }_{q}={\varepsilon }_{N\alpha }=\hslash {\omega }_{c}(N+\mathrm{1/2}+\zeta \alpha )$ as the energy spectrum in equation (1)^7–10^. Here ${\omega }_{c}=eB/Mc$ is the cyclotron frequency, and *M* is the electron effective mass. The quantum number *N* is a non-negative integer. The spin variable *α* takes the values ±1. The Zeeman spin term is $\hslash {\omega }_{c}\zeta \alpha$ with $\zeta =({g}^{\ast }/\mathrm{2)(}M\mathrm{/2}{M}_{0})$, where *M*_0_ is the electron rest mass. The degeneracy of a Landau level with a given spin variable is $D(N,\alpha )=eB/hc\equiv {D}_{0}\mathrm{.}$ The magnetic induction *B* in this *D*_0_ cancels the *B*-dependence of the factor *ecB*^−1^ in equation (1). Consequently, the inverse of Hall resistance for IQHE is

2 $${R}_{{\rm{H}}}^{-1}={e}^{2}{h}^{-1}\sum _{N=0}^{\infty }\sum _{\alpha }{\{1+\exp [({\varepsilon }_{N\alpha }-\mu )/{k}_{B}T]\}}^{-1}\mathrm{.}$$

In the zero temperature limit the inverse of Hall resistance becomes

3 $$\mathop{\mathrm{lim}}\limits_{T\to 0}\,{R}_{{\rm{H}}}^{-1}={e}^{2}/h\sum _{N=0}^{\infty }\sum _{\alpha }\theta ({B}_{N\alpha }-B),\,\,{B}_{N\alpha }=(\mu Mc/e\hslash )\{N+\mathrm{1/2}+\alpha \zeta {\}}^{-1}\mathrm{.}$$

This calculation shows the quantisation unit of ${R}_{{\rm{H}}}^{-1}$ is *e*^2^/*h* because of the degeneracy of a Landau level *D*_0_. That is,

4 $$ec{B}^{-1}{D}_{0}=ec{B}^{-1}(eB/hc)={e}^{2}/h\mathrm{.}$$

Considering the above analysis, let us inspect the Hall resistance data in the FQHE experiment reported in ref.^14^. The quantisation unit of ${R}_{{\rm{H}}}^{-1}$ on *e*^2^/3*h*, 2*e*^2^/3*h* and 4*e*^2^/3*h* plateaus observed in the FQHE experiment^14^ is *e*^2^/3*h*. In view of equation (4) the most plausible explanation for this is that a Landau level is split into three sublevels. Each sublevel has the degeneracy *D*_1_ = *D*_0_/3. We assume that the level-splitting is caused by a perturbation Hamiltonian ${ {\mathcal H} }_{1}^{^{\prime} }$, which yields new quantum numbers *m*_1_ = −1, 0, 1 for sublevels. Let us call these sublevels the *m*_1_ sublevels. The 2*e*^2^/5*h*, 2*e*^2^/5*h*, 4*e*^2^/5*h*, and 7*e*^2^/5*h* plateaus in FQHE can be explained by assuming an additional perturbation Hamiltonian ${ {\mathcal H} }_{2}^{^{\prime} }$ that splits each *m*_1_ sublevel into five sublevels. Let us call these sublevels the *m*_2_ sublevels. Each sublevel has the degeneracy *D*_2_ = *D*_1_/5.We assume that ${ {\mathcal H} }_{2}^{^{\prime} }$ is small perturbation to ${ {\mathcal H} }_{1}^{^{\prime} }$. The 3*e*^2^/7*h* and 4*e*^2^/7*h* plateaus in FQHE can be explained by assuming an additional perturbation Hamiltonian ${ {\mathcal H} }_{3}^{^{\prime} }$ that splits each *m*_2_ sublevel into seven sublevels. Let us call these sublevels the *m*_3_ sublevels. Each sublevel has the degeneracy *D*_3_ = *D*_2_/7.We assume that ${ {\mathcal H} }_{3}^{\text{'}}$ is small perturbation to ${ {\mathcal H} }_{2}^{\text{'}}$. Hence, the quantised values of FQHE resistance at fractional plateaus can be attributed to the degeneracies of sequentially split sublevels. This analysis indicates a model energy spectrum

5 $$\varepsilon (N,\alpha ,m)={\varepsilon }_{N\alpha }+\hslash {\omega }_{c}\sum _{l=1}^{{l}_{{\rm{\max }}}}{\lambda }_{l}{m}_{l},$$

where *m*_*l*_ is an integer ranging $-l\le {m}_{l}\le l$. We have defined *m* = (*m*_1_, *m*_2_, *m*_3_, …). The parameters *λ*_*l*_ are assumed to satisfy the condition |*λ*_*l*+1_| < |*λ*_*l*_|. Using the Hall resistance formula given by equation (1), we can determine the parameters *λ*_*l*_ from the experiment. If *λ*_*l*_ are independent of *B*, in the zero-temperature limit, the locations of step edges on the *B* axis are given by equation (3) as

6 $${B}_{N\alpha m}^{{\rm{F}}{\rm{Q}}{\rm{H}}{\rm{E}}}=(\mu Mc/e\hslash ){[N+1/2+\alpha \zeta +\sum _{l=1}^{{l}_{max}}{\lambda }_{l}{m}_{l}]}^{-1}.$$

By reading the values of *B*_*Nα*_ from the experimental Hall resistance data at very low temperatures, it is possible to determine *λ*_*l*_.

## Results

### Odd-denominator fractional plateaus

Because the number of possible *m*_*l*_’s for a given *l* is 2*l* + 1, the degeneracy of an energy level with quantum numbers (*N*, *α*, *m*) is $D(N,\alpha ,m)={D}_{0}(N,\alpha ){\prod }_{l=1}^{{l}_{{\rm{\max }}}}{\mathrm{(2}l+\mathrm{1)}}^{-1}$. Hence the inverse of Hall resistance for FQHE is given as

7 $${R}_{{\rm{H}}}^{-1}={e}^{2}{h}^{-1}\sum _{N=0}^{\infty }\sum _{\alpha }\prod _{l=1}^{{l}_{{\rm{\max }}}}{\mathrm{(2}l+\mathrm{1)}}^{-1}\sum _{m}{\{1+\exp [(\varepsilon (N,\alpha ,m)-\mu )/{k}_{B}T]\}}^{-1},$$

where we have defined ${\sum }_{m}={\sum }_{{m}_{1}}\,{\sum }_{{m}_{2}}\cdot \cdot \cdot {\sum }_{{m}_{{l}_{{\rm{\max }}}}}$. This formula yields the values of Hall resistance on plateaus as

8 $${R}_{{\rm{H}}}^{-1}=\frac{{e}^{2}}{h}\frac{j}{\prod _{l=1}^{{l}_{max}}(2l+1)}\,\,(j=1,\,2,\,\ldots )\,\,,$$

where *j* is a positive integer. This formula yields all 75 observed odd-denominator fractional plateaus^15,16^. It should be noted that the Hall resistance given by equation (8) is consistent with the result obtained on the basis of the fractal geometry^17^.

### Magnetic induction dependence of Hall resistance

For the practical purpose of calculating the magnetic induction dependence of Hall resistance we assume *l*_max_ = 3 in the model energy spectrum given by equation (5) such that

9 $$\varepsilon (N,\alpha ,m)={\varepsilon }_{N\alpha }+\hslash {\omega }_{c}\{{\lambda }_{1}{m}_{1}+{\lambda }_{2}{m}_{2}+{\lambda }_{3}{m}_{3}\}\mathrm{.}$$

**Figure 1:**
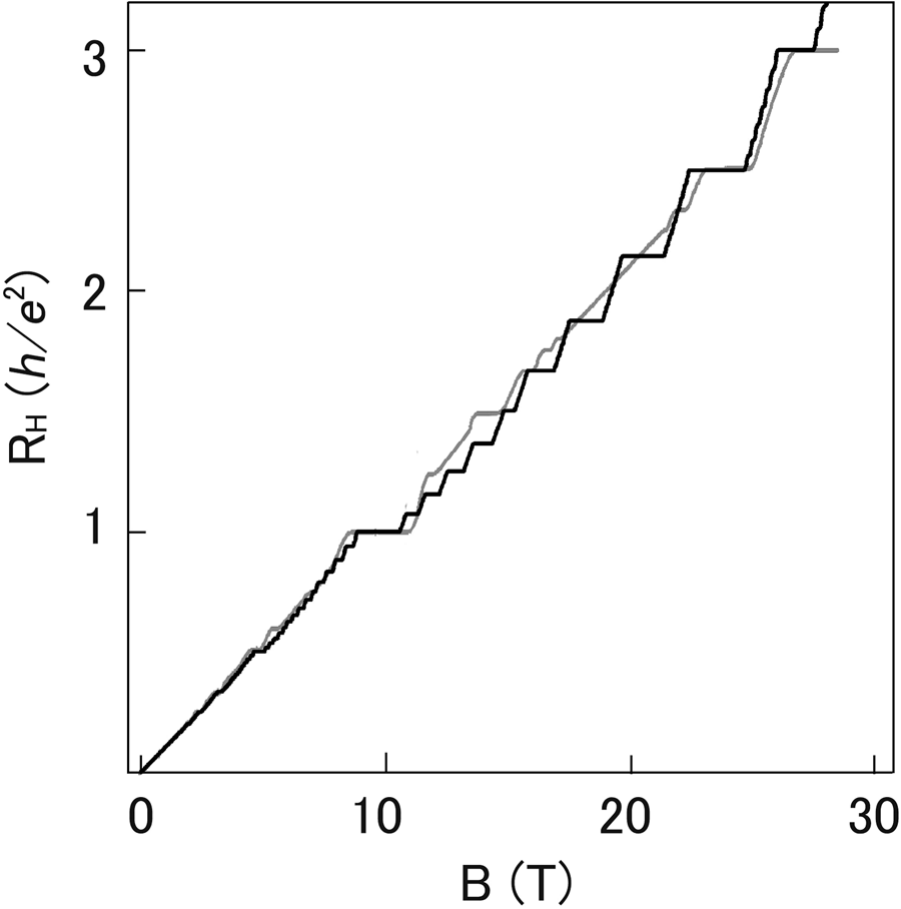
Theoretical Hall resistance as a function of magnetic induction. Hall resistance at *T* = 85 mK calculated by using equation (7) is plotted in black. The ordinate is Hall resistance in units of the von Klitzing constant $h/{e}^{2}\simeq 25812.8$ ohm. The experimental Hall resistance at *T* = 85 mK from ref.^14^ is also plotted in gray.

**Figure 2:**
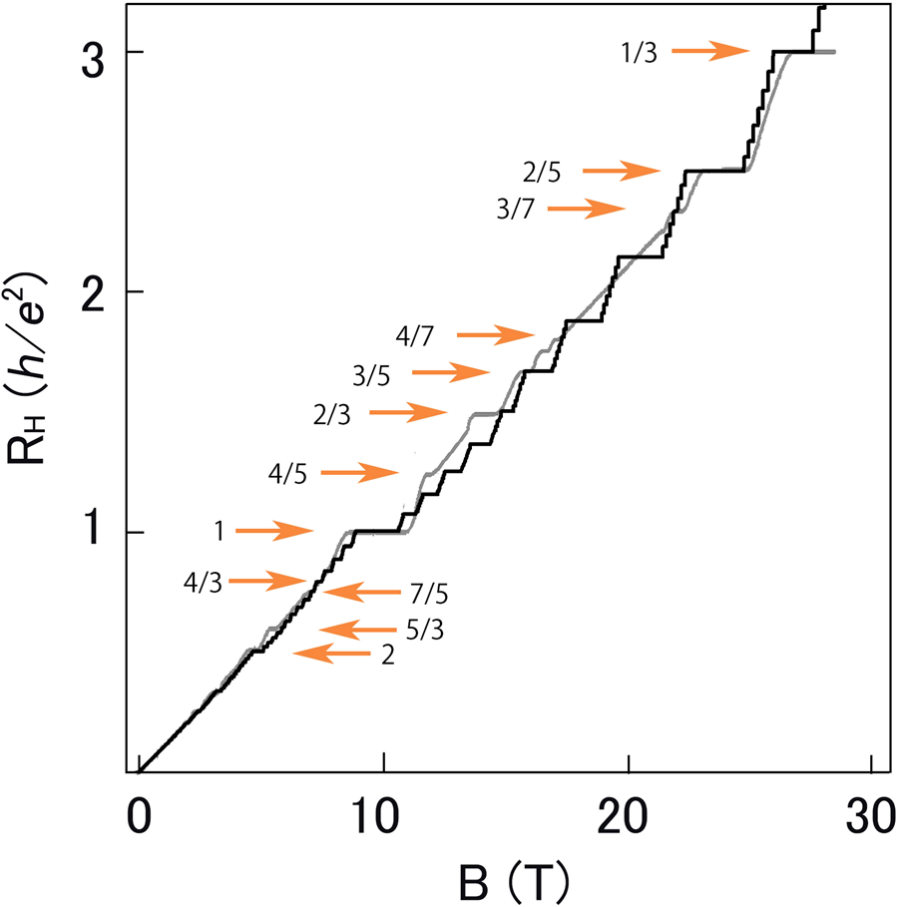
Theoretical Hall resistance as a function of magnetic induction. Hall resistance at *T* = 5 mK calculated by using equation (7) is plotted in black. The ordinate is Hall resistance in units of the von Klitzing constant $h/{e}^{2}\simeq 25812.8$ ohm. The experimental Hall resistance at *T* = 85 mK from ref.^14^ is also plotted in gray. The horizontal arrows indicate plateaus.

**Figure 3:**
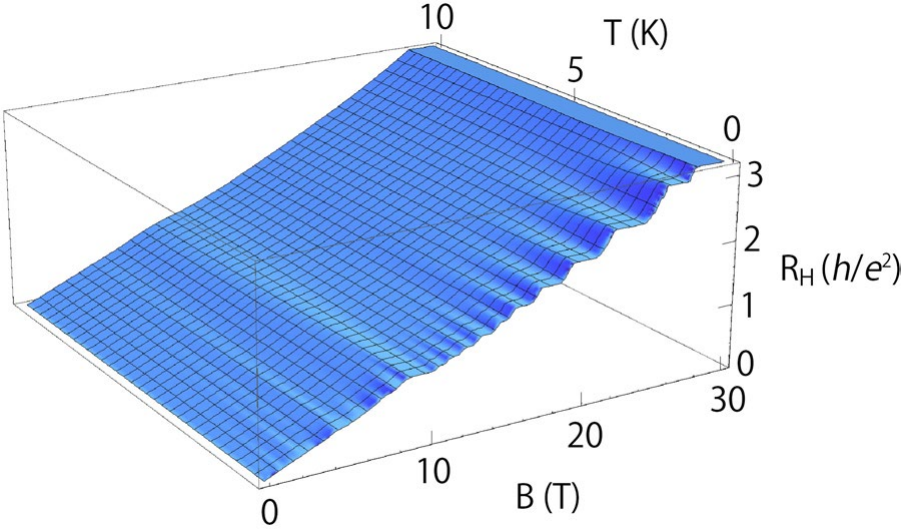
Theoretical Hall resistance as a function of *B* and *T*. Hall resistance calculated by using equation (7) is shown as a 3D plot for 0 < *T* < 10 K and 0 < *B* < 30 T. The ordinate is Hall resistance in units of the von Klitzing constant $h/{e}^{2}\simeq 25812.8$ ohm.

Note that the parameters *λ*_*l*_ may depend on *B*. For instance if we assume ${\lambda }_{j}\propto \mathrm{1/}\sqrt{B}$ then the energy gaps corresponding to the fractional plateaus become proportional to $\sqrt{B}$. Here we consider the simplest case that *λ*_*l*_ are independent of *B*. Then the three parameters *λ*_*l*_ in equation (9) are fitted to the experimental Hall resistance curve in ref.^14^. Their values are *λ*_1_ = 0.25, *λ*_2_ = 0.14, and *λ*_3_ = 0.003. Considering the Hall resistance data for the IQHE experiment in ref.^18^, the effective g-factor is adjusted to ${g}^{\ast }=12$. The effective mass is *M* = 0.067 *M*_0_. The chemical potential is determined by the slope of experimental Hall resistance curve for weak magnetic induction. The value is $\mu =13.14\times {10}^{-15}$ erg. The theoretical resistance curve as a function of *B* is plotted in Fig. 1, using equations (7) and (9) for *T* = 85 mK which is the experimental temperature in ref.^14^. In order to see the plateaus clearly the theoretical resistance curve for *T* = 5 mK is plotted in Fig. 2. The theoretically calculated Hall resistance plateaus 1/3, 2/5, 3/7, 4/7, 3/5, 2/3, 4/5, 1, 4/3, 7/5, 5/3, and 2 are consistent with the experiment^14^. Although the theoretical curve agrees with experiment fairly well, it seems necessary to consider the *B*-dependence of *λ*_*l*_ to improve agreement. In Fig. 3 the magnetic induction and temperature dependence of the Hall resistance is shown in a 3D plot. It shows the Hall resistance curve given by equation (7) becomes classical as temperature increases. Hence the formula (7) can yield FQHE, IQHE and classical Hall effects.

## Discussion

The quantum number *m*_*l*_ introduced in the model perturbation energy spectrum (5) ranges $-l\le {m}_{l}\le l$. Therefore, it is plausible that these quantum numbers *m*_*l*_ and *l* correspond to angular momentum. Because the orbital angular momentum operators cannot be defined in the 2-dimensional space, it is necessary to consider the problem in the 3-dimensional space. We adopt $(r,\theta ,\varphi )$ for the 3-dimensional polar coordinates. Then the 3-dimensional lowest Landau level (LLL) wave function is^19^

10 $${{\rm{\Phi }}}_{0}^{3{\rm{D}}}={N}_{m}{(\frac{{d}^{2}}{{a}^{2}})}^{|m|/2}\exp (\,-\,{z}^{2})\sum _{j=0}\frac{1}{j!}{(1-\frac{{d}^{2}}{2{a}^{2}})}^{j}\frac{{({z}^{2})}^{(|m|+2j)/2}}{\{2(|m|+2j)-1\}!!}\sum _{l=0}^{{\rm{\infty }}}C(l,m;j){Y}_{lm}(\theta ,\varphi ),$$

where *N*_*m*_ is the normalisation factor, $a=\sqrt{c\hslash /eB}$ is the magnetic length, *Y*_*lm*_ is spherical harmonics, *d* is the thickness of the 2-dimensional system, and ${z}^{2}={r}^{2}\mathrm{/2}{d}^{2}$. The expansion (10) shows that the lowest Landau level in the three-dimensional space is a superposition of angular momentum eigenstates of different *l*. The allowed values of *m* in equation (10) are only non-positive integers^19^. Because the quantum number *m*_*l*_ ranges from *l* to −*l*, it cannot belong to the unperturbed state given by equation (10). Therefore, the quantum number *m*_*l*_ possibly corresponds to new degree of freedom of the Landau orbitals in the three-dimensional space.

A model based on magnetoplasmon excitations in the three-dimensional space may explain the energy spectrum given by equation (5). The experimentally observed quantised plateaus in the magnetic induction dependence of magnetoplasmon dispersion^20^ clearly indicate significance of magnetoplasmons in the quantum Hall effects^6,9^. In the fractional quantum Hall regime the electron system can be regarded as a liquid of electrons in LLL orbitals. In order to examine how the magnetoplasmon fields affect the dynamics of a single-electron energy spectrum, we assume an electron P in a LLL orbital whose center is located at the origin of the coordinates. The Maxwell equations for magentoplasmon fields have source terms due to the electrons and uniform positive background^13^. In the following discussions we shall use the Lorentz gauge and four vector notations for simplicity of mathematical expressions and to avoid the decomposition of vector fields into transverse and longitudinal components^21,22^. Then the current densities in source terms in the Maxwell equations can be written as

11 $${j}_{\mu }(x)={j}_{\mu }^{{\rm{P}}}(x)+{j}_{\mu }^{{\rm{induced}}}(x),$$

where ${j}_{\mu }^{{\rm{P}}}$ is due to the electron P and ${j}_{\mu }^{{\rm{induced}}}$ is due to the other electrons. The Greek subscripts denote the components in the four-dimensional space^23^. The background uniform charge is included in these terms. In the self-consistent linear response approximation (SCLRA)^6,21,24,25^ the latter is given as

12 $${j}_{\mu }^{{\rm{induced}}}(x)=\int {d}^{4}x^{\prime} \sum _{\nu =0}^{3}{{\rm{\Lambda }}}_{\mu \nu }(x,x^{\prime} ){A}_{\nu }^{{\rm{mp}}}(x^{\prime} ),$$

where ${{\rm{\Lambda }}}_{\mu \nu }(x,x^{\prime} )$ is the retarded current-current response function^21,26^ of the electron system. The Maxwell equations for magentoplasmon fields in the SCLRA can be written

13 $$({\nabla }^{2}-{c}^{-2}{\partial }_{t}^{2}){A}_{\mu }^{{\rm{mp}}}(x)+4\pi {c}^{-1}\int {d}^{4}x^{\prime} \sum _{\nu =0}^{3}{{\rm{\Lambda }}}_{\mu \nu }(x,x^{\prime} ){A}_{\nu }^{{\rm{mp}}}(x^{\prime} )=-\,4\pi {c}^{-1}{j}_{\mu }^{{\rm{P}}}(x\mathrm{).}$$

We consider magnetoplasmons whose wavelengths are much larger than Landau radius. Then, the current density ${j}_{\mu }^{{\rm{P}}}$ is well localised in the vicinity of the origin of the coordinates. This type of equations with a localised source term has been intensively studied in the theories of multipole fields such as radiation of electromagnetic fields^27,28^, and it is known that spherical harmonics *Y*_*lm*_(*θ*, *ϕ*) are most relevant orthogonal basis to expand the field variables, particularly stationary waves. Therefore, the stationary modes of magentoplasmons given by equation (13) are labeled with (*l*, *m*). The propagator for the quantised magnetoplasmon field can be calculated from equation (13). Then the self-energy of the temperature Green function for the electron field can be expressed in terms of the magnetoplasmon propagator by virtue of the finite temperature generalised Ward-Takahashi relations^12,29^. Consequently, the electron energy spectrum will acquire perturbation terms labeled with (*l*, *m*).

The essential features of the SCLRA equations are determined by the retarded current-current response functions of the many-electron system^21,26^. Therefore, in order to elaborate on this magnetoplasmon model of the spectrum given by equation (5) it is necessary to calculate these response functions, or to investigate their mathematical properties. The physical idea of this magnetoplasmon model is very similar to that of the Lamb shift in quantum electrodynamics^30,31^. While an electron bound to an atomic orbital interacts with electromagnetic field in the Lamb shift, here an electron in LLL orbital interacts with magnetoplasmon. In both cases the reaction of the electromagnetic fields is the essential cause of the phenomena. Although magnetoplasmon is electromagnetic field, its modes are much more complicated than the electrogamgnetic field in the Lamb shift.

Since the discovery of the fractional quantum Hall effect, there have been a number of interesting theoretical models^32^. Among them the fractal geometry model^17^ seems to be deeply related to the present model. The Hall resistance formula given by equation (8) is consistent with the results given in ref.^17^. It is an interesting theoretical problem to investigate whether the current-current response functions can contain a geometrical structure such as the fractal geometry discussed in ref.^17^.

We explained the fractional quantised values of the Hall resistance on plateaus in terms of the degeneracies of sublevels created from Landau levels by the phenomenologically introduced perturbation in the single-electron energy spectrum. The present theory yields all 75 odd-denominator fractional values observed experimentally to date^15,16^. The simple model with *l*_***m****ax*_ = 3 yields twelve plateaus whose magnetic induction dependence is consistent with the experiment. No existing theories can yield this quantitative fit to the experiment. By calculating the temperature dependence of the Hall resistance, we plotted a 3D graph that explicitly shows how FQHE and IQHE disappear and become classical Hall effect as temperature increases. Because the Hall resistance formula (1) depends only on the single-electron energy spectrum via Fermi distributions and can explain both IQHE and FQHE, this theory clearly shows that the Fermi liquid theory^11,12,33^ is valid for IQHE and FQHE.

## Methods

### Derivation of Hall resistance formula

The *x*_1_ axis is taken along the direction of the bias current, and the *x*_3_ axis is taken along the direction of the magnetic field. Magnetic induction and electric field are given as ***B*** = (0, 0, *B*) and ${{\boldsymbol{E}}}^{i}=({E}_{1}^{i},{E}_{2}^{i},\,\mathrm{0)}$, respectively. The effects of the Lorentz force on the electron currents can be calculated using the Heisenberg equations for the mechanical momentum operators

14 $${P}_{k}^{i}={\int }_{{{\rm{\Omega }}}^{i}}{\psi }_{\alpha }^{\dagger }(x)\{\,-\,i\hslash {\partial }_{k}+e{c}^{-1}{A}_{k}\}{\psi }_{\alpha }(x),$$

where *ψ*_*α*_ and ${\psi }_{\alpha }^{\dagger }$ are the second quantized electron field operators in the Heisenberg picture. The integral notation is defined as ${\int }_{{{\rm{\Omega }}}^{i}}={\int }_{0}^{L}d{x}_{1}{\int }_{0}^{{\rm{\Delta }}L}d{x}_{2}$, where *L* and Δ*L* are the length and width of a subsystem Ω^*i*^. We use Einstein convention for the summation over the spin variables. The superscript *i* denotes a subsystem. We also define the electron density operator $\rho ={\psi }_{\alpha }^{\dagger }{\psi }_{\alpha }$ and the electric current density operator ${J}_{k}=(\,-\,e/M){\psi }_{\alpha }^{\dagger }\{-i\hslash {\partial }_{k}+(e/c){A}_{k}\}{\psi }_{\alpha }$. The electron effective mass is denoted by *M*. Noting that the equal-time canonical commutators of ${P}_{k}^{i}$ with *H*_spin_, *H*_e_, and *H*_int_ simply vanish^10^, i.e. $[{P}_{k}^{i},{H}_{{\rm{spin}}}]=[{P}_{k}^{i},{H}_{{\rm{e}}}]=[{P}_{k}^{i},{H}_{{\rm{int}}}]=0$, we can readily calculate the Heisenberg equations and find

15 $${{\rm{\partial }}}_{t}{P}_{1}^{i}=(\,-\,eB/Mc){P}_{2}^{i}-e{\int }_{{{\rm{\Omega }}}^{i}}{E}_{1}^{i}\rho ,\,\,\,{{\rm{\partial }}}_{t}{P}_{2}^{i}=(eB/Mc){P}_{1}^{i}-e{\int }_{{{\rm{\Omega }}}^{i}}{E}_{2}^{i}\rho .$$

Because the mechanical momentum operators and gauge current density operators satisfy the relation ${P}_{k}^{i}=-\,m{e}^{-1}{\int }_{{{\rm{\Omega }}}^{i}}{J}_{k}$, equations (15) give the equations of motion for the current density operators^10^. The next steps are to take the statistical mechanical ensemble average of each term in the equations and to introduce a phenomenological damping terms ${W}_{k}^{i}$, which are necessary to ensure the Ohm’s law. We obtain

16 $${\partial }_{t}{\int }_{{{\rm{\Omega }}}^{i}}{\langle {J}_{1}\rangle }^{i}=(\,-\,eB/Mc){\int }_{{{\rm{\Omega }}}^{i}}{\langle {J}_{2}\rangle }^{i}+({e}^{2}/M){\int }_{{{\rm{\Omega }}}^{i}}{E}_{1}^{i}{\langle \rho \rangle }^{i}+{W}_{1}^{i},$$

and

17 $${\partial }_{t}{\int }_{{{\rm{\Omega }}}^{i}}{\langle {J}_{2}\rangle }^{i}=(eB/Mc){\int }_{{{\rm{\Omega }}}^{i}}{\langle {J}_{1}\rangle }^{i}+({e}^{2}/M){\int }_{{{\rm{\Omega }}}^{i}}{E}_{2}^{i}{\langle \rho \rangle }^{i}+{W}_{2}^{i}\mathrm{.}$$

where ${\langle \mathrm{...}\rangle }^{i}$ denotes the statistical ensemble average in a subsystem Ω^*i*^. The damping terms must vanish if there are no currents. Therefore they must satisfy the condition^33^ ${W}_{k}^{i}=0$ for ${\int }_{{{\rm{\Omega }}}^{i}}{\langle {J}_{k}\rangle }^{i}=0$. Considering the experimental conditions, we impose the steady state condition ${\partial }_{t}{\int }_{{{\rm{\Omega }}}^{i}}{\langle {J}_{k}\rangle }^{i}=0$ and the condition ${\int }_{{{\rm{\Omega }}}^{i}}{\langle {J}_{2}\rangle }^{i}=0$, which gives *W*_2_ = 0. Consequently equation (17) yields

18 $$0=(eB/Mc){\int }_{{{\rm{\Omega }}}^{i}}{\langle {J}_{1}\rangle }^{i}+({e}^{2}/M){\int }_{{{\rm{\Omega }}}^{i}}{E}_{2}^{i}{\langle \rho \rangle }^{i}\mathrm{.}$$

Although the electron-electron interaction *H*_int_ is rigorously included in the derivation, the electron-electron potential does not appear explicitly in this equation. The details of the damping term *W*_2_ are not necessary. The only required condition for the damping terms is that they must vanish when there is no current.

To calculate the Hall resistance it is necessary to define macroscopic currents *I*_*k*_ that correspond to experimentally measurable currents. We assume <*ρ*>^*i*^, <*J*_1_>^*i*^ and <*J*_2_>^*i*^ in each subsystem are uniform. We first define macroscopic currents ${I}_{k}^{i}$ in a subsystem Ω^*i*^ such that

19 $${\int }_{{{\rm{\Omega }}}^{i}}{\langle {J}_{1}\rangle }^{i}={L}_{1}{\rm{\Delta }}L{\langle {J}_{1}\rangle }^{i}={L}_{1}{I}_{1}^{i},\,\,\,{\int }_{{{\rm{\Omega }}}^{i}}{\langle {J}_{2}\rangle }^{i}={L}_{1}{\rm{\Delta }}L{\langle {J}_{2}\rangle }^{i}={\rm{\Delta }}L{I}_{2}^{i}.$$

We also define the Hall voltage in each subsystem such that ${V}_{2}^{i}=-\,{E}_{2}^{i}{\rm{\Delta }}L$. Then we have ${I}_{1}^{i}=(ec/B){V}_{2}^{i}{\rho }^{i}$, which holds for each subsystem. By adding ${I}_{1}^{i}$ from all subsystems, we obtain ${I}_{1}=(ec/B){\sum }_{i}{V}_{2}^{i}{\rho }^{i}$, where ${I}_{1}={\sum }_{i}{I}_{1}^{i}$ is the experimentally measurable macroscopic current. We assume the expectation value for the electron number density is given in terms of the Fermi distribution function $f({\varepsilon }_{q}+\delta {\varepsilon }^{i};T)={[1+\exp \{({\varepsilon }_{q}+\delta {\varepsilon }^{i}-\mu )/{k}_{B}T\}]}^{-1}$. The electron energy spectrum in the subsystem Ω^*i*^ consists of an *i*-independent part *ε*_*q*_ and an *i*-dependent part *δε*_*i*_, where *q* is the quantum number of a quasi-electron state. The total current *I*_1_ is ${I}_{1}=ec{B}^{-1}{\sum }_{i}{V}_{2}^{i}{\sum }_{q}D(q)f({\varepsilon }_{q}+\delta {\varepsilon }^{i};T)$, where *D*(*q*) is the degeneracy of the energy level *q*. The experimentally measurable Hall potential difference is ${V}_{2}={\sum }_{i}{V}_{2}^{i}$. In general the presence of *δε*^*i*^ in the Fermi distribution prohibits the evaluation of the sum over *i* to obtain *V*_2_/*I*_1_. However, if *δε*^*i*^ is much smaller than the smallest increment of energy level *ε*_*q*_, then the summation of the Fermi distributions is possible. After the summation over *i* we find ${I}_{1}=ec{B}^{-1}{V}_{2}{\sum }_{q}D(q)f({\varepsilon }_{q};T)$. This yields the inverse of Hall resistance

20 $${R}_{{\rm{H}}}^{-1}=ec{B}^{-1}\sum _{q}D(q){\{1+\exp [({\varepsilon }_{q}-\mu )/{k}_{B}T]\}}^{-1}\mathrm{.}$$

The single-electron energy spectrum is denoted by *ε*_*q*_ with a quantum number *q*. The degeneracy of energy level *q* is denoted by *D*(*q*).

### Expansion coefficients in the 3-dimensional lowest Landau level wave function

The quantum number *m*_*l*_ introduced in the model perturbation energy spectrum given in equation (5) ranges $-l\le {m}_{l}\le l$. Therefore, it is plausible that these quantum numbers *m*_*l*_ and *l* correspond to angular momentum. Because the orbital angular momentum operators cannot be defined in the 2-dimensional space, it is necessary to consider the problem in the 3-dimensional space. We adopt the vector potential ***A*** = (−*Bx*_2_/2, *Bx*_1_/2, 0). Then the lowest Landau level wave function is^19^

21 $${{\rm{\Phi }}}_{0}^{2{\rm{D}}}=\frac{1}{\sqrt{2\pi }}{N}_{0,m}^{2{\rm{D}}}\,\exp (im\varphi )\exp (\frac{-\xi }{2}){\xi }^{|m|/2},\,\,\,\xi =\frac{{\rho }^{2}}{2{a}^{2}}=\frac{{r}^{2}{\sin }^{2}\theta }{2{a}^{2}}.$$

where ${N}_{\mathrm{0,}m}^{{\rm{2D}}}$ is the normalisation factor, and $a=\sqrt{c\hslash /eB}$ is the magnetic length. Here we use (*ρ*, *ϕ*) for the 2-dimensional polar coordinates and (*r*, *θ*, *ϕ*) for the 3-dimensional polar coordinates. It is also necessary to consider explicitly the confining potential *V*_conf._ (*x*_3_) and the 3-dimensional kinetic energy in the Hamiltonian^26,34^. We assume the electrons are in the ground state of *V*_conf._ (*x*_3_) with a simple Gaussian wave function ${\chi }_{0}={(\sqrt{2\pi }d)}^{-1}\exp [-{r}^{2}{\cos }^{2}\theta \mathrm{/2}{d}^{2}]$, where *d* is the thickness of the 2-dimensional system. The product of ${{\rm{\Phi }}}_{0}^{{\rm{2D}}}$ and *χ*_0_ yields the 3-dimensional lowest Landau level wave function

22 $${{\rm{\Phi }}}_{0}^{3{\rm{D}}}={N}_{m}{(\frac{{d}^{2}}{{a}^{2}})}^{|m|/2}\exp (\,-\,{z}^{2})\sum _{j=0}\frac{1}{j!}{(1-\frac{{d}^{2}}{2{a}^{2}})}^{j}\frac{{({z}^{2})}^{(|m|+2j)/2}}{\{2(|m|+2j)-1\}!!}\sum _{l=0}^{{\rm{\infty }}}C(l,m;j){Y}_{lm}(\theta ,\varphi ),$$

where *N*_*m*_ is the normalisation factor, *Y*_*lm*_ is spherical harmonics and *z*^2^ = *r*^2^/2*d*^2^. The expansion coefficient *C*(*l*, *m*; *j*) is calculated as

23 $$C(l,m;j)=\sqrt{2\pi }{[\frac{2l+1}{2}\frac{(l-|m|)!}{(l+|m|)!}]}^{1/2}{\int }_{-1}^{1}dx{P}_{l}^{|m|}(x){P}_{|m|+2j}^{|m|+2j}(x)=\sqrt{2\pi }{[\frac{2l+1}{2}\frac{(l-|m|)!}{(l+|m|)!}]}^{1/2}I(l,|m|,j)\,.$$

This integral *I*(*l*, |*m*|, *j*) can be analytically evaluated^35^. If *l* + |*m*| is even and $(l-|m\mathrm{|)/2}\le j$, then

24 $$I(l,|m|,j)=2(\,-\,1{)}^{(l-|m|)/2}\frac{(2|m|+4j-1)!!(2|m|+2j)!!(l+|m|-1)!!}{(l+|m|+2j+1)!!}(\begin{array}{c}j\\ \frac{l-|m|}{2}\end{array}).$$

If *l* + |*m*| is even and $j < (l-|m|)/2$, then *I*(*l*, |*m*|, *j*) = 0. If *l* + |*m*| is odd, then *I*(*l*, |*m*|, *j*) = 0.
